# The Intracellular Domain of Dumbfounded Affects Myoblast Fusion Efficiency and Interacts with Rolling Pebbles and Loner

**DOI:** 10.1371/journal.pone.0009374

**Published:** 2010-02-23

**Authors:** Sarada Bulchand, Sree Devi Menon, Simi Elizabeth George, William Chia

**Affiliations:** Temasek Lifesciences Laboratory, National University of Singapore, Singapore, Singapore; Fred Hutchinson Cancer Research Center, United States of America

## Abstract

Drosophila body wall muscles are multinucleated syncytia formed by successive fusions between a founder myoblast and several fusion competent myoblasts. Initial fusion gives rise to a bi/trinucleate precursor followed by more fusion cycles forming a mature muscle. This process requires the functions of various molecules including the transmembrane myoblast attractants Dumbfounded (Duf) and its paralogue Roughest (Rst), a scaffold protein Rolling pebbles (Rols) and a guanine nucleotide exchange factor Loner. Fusion completely fails in a *duf, rst* mutant, and is blocked at the bi/trinucleate stage in *rols* and *loner* single mutants. We analysed the transmembrane and intracellular domains of Duf, by mutating conserved putative signaling sites and serially deleting the intracellular domain. These were tested for their ability to translocate and interact with Rols and Loner and to rescue the fusion defect in *duf, rst* mutant embryos. Studying combinations of double mutants, further tested the function of Rols, Loner and other fusion molecules. Here we show that serial truncations of the Duf intracellular domain successively compromise its function to translocate and interact with Rols and Loner in addition to affecting myoblast fusion efficiency in embryos. Putative phosphorylation sites function additively while the extreme C terminus including a PDZ binding domain is dispensable for its function. We also show that fusion is completely blocked in a *rols, loner* double mutant and is compromised in other double mutants. These results suggest an additive function of the intracellular domain of Duf and an early function of Rols and Loner which is independent of Duf.

## Introduction

Skeletal muscles perform various roles, which include coordinating movement and stabilising joints in many organisms. Understanding how they develop has been the focus of several studies [1]. Studying this process in vertebrate models is complicated by the relative inaccessibility of their muscles and long developmental times. Simple model organisms like *Drosophila melanogaster* have been widely used instead. Its somatic/body wall muscles (analogous to vertebrate skeletal muscles) are easily accessible and several muscle specific genes are conserved with those of vertebrates [2]. Also, some principles of muscle development are similar [3], [4]. *Drosophila* somatic muscles develop during mid to late embryogenesis, display their contractile function during late embryogenesis and continue to function in the developing larva where they are critical for motility [5], [6].

During early embryogenesis, two types of myoblasts are specified, the founder cells (FCs) and the fusion competent myoblasts (FCMs) [7], [8]. The FCs express the myoblast attractants, Dumbfounded (Duf)/Kin of irregular-chiasm-C (Kirre) and its paralogue Roughest (Rst)/Irregular chiasm-C (IrreC) [9], [10]. They also express muscle identity genes, like Even-skipped (Eve) [11], that are responsible for the specification of muscle size, position with respect to the body axis, points of epidermal attachment and points of innervation [8], [12]. The FCMs on the other hand constitute a more homogeneous population of cells expressing the Duf/Rst ligands, Sticks and Stones (SNS) [13] and Hibris (Hbs) [14]. The FCMs contribute to muscle size by fusing with the FCs [8], [15]. Fusion always occurs between FC/myotube and FCM and never between cells of the same type [16].

Recent studies have shown that myoblasts are spatially organised in the embryo. Fusion initiates between an FC and FCM that are in its vicinity. As development proceeds, FCMs appear to migrate towards the FC for further rounds of fusion [17]. Duf/Rst expressed on the FC surface and SNS/Hbs expressed on the FCM surface are thought to bring about myoblast attraction, and have been suggested to actively participate in this process [9], [14], [18], [19]. Upon FC-FCM contact and adhesion, the plasma membranes breakdown leading to cytoplasmic continuity [4]. The presence of vesicles and electron dense plaques at the site of adhesion prior to membrane breakdown have also been observed but the nature and content of these vesicles are unknown [20]. Further studies have revealed the accumulation of an F actin focus (FuRMAS) at the site of myoblast adhesion [21] and live imaging data indicate that the F-actin focus marks the site of fusion [22]. Proteins like Duf and SNS localise to this focus suggesting that this might be their site of activity during fusion [21]. Upon fusion, the nuclei of the FCMs are entrained by the FC nucleus and begin to express FC specific molecules [6]. The process of fusion is reiterative. Events are repeated in a stepwise manner first leading to the formation of a bi/trinucleate precursor, followed by more such rounds of fusion, accompanied by growth at the ends of the myotube. As embryogenesis proceeds the newly formed muscles attach to specific sites at the epidermis leading to the formation of approximately 30 muscles per hemisegment [8].

Genetic screens have identified a large number of molecules required for myoblast fusion that fall into several categories depending on their predicted functions [23], [24]. Mutation of these genes, in most cases, leads to the formation of defective “mini muscles” with reduced nuclei, ending in embryonic lethality. Duf and Rst are Type I single pass transmembrane receptors with an N terminal extracellular domain and C terminal intracellular domain, belonging to the Immunoglobulin superfamily of proteins [10], [25]. Their function is redundant in the FC. In mutant embryos that lack both *duf* and *rst*, *Df(1)w^67k30^* (henceforth called the *duf, rst* mutant), there is no attraction and adhesion between FCs and FCMs leading to a complete block in fusion [9], [10]. Both the extracellular and intracellular domains of Duf have been shown to be critical for the attraction of FCMs and sustenance of fusion respectively [25]. In the absence of the extracellular domain FCMs are not attracted towards the FC and fusion fails. In the absence of the intracellular domain fusion is not sustained beyond the first phase, stalling at the bi/tri nucleate precursor stage [25]. This suggests that the intracellular domain might interact with proteins that function to sustain fusion.

Previous studies have shown that Rolling pebbles (Rols)/Antisocial (Ants), a scaffold protein with multiple protein interaction domains, is involved in sustaining fusion beyond the bi/trinucleate precursor stage. Fusion in *rols* mutant embryos stalls at this precursor stage [26]–[28]. On the other hand, Loner, an Arf6 guanine nucleotide exchange factor (GEF), has been reported to be involved in the initial stage of fusion with minimal fusion occurring in a *loner* single mutant. However, binucleate precursors are observed [17], [29]. Rols and Loner have been shown to respond to Duf and translocate to points of cell contact in a Duf dependent manner in transfected S2 cells and in the case of Rols, in embryos as well [25], [29]. Both Rols and Loner colocalise with Duf but do not colocalise with each other suggestive of functions in different pathways [25], [29]. While it has been shown that Duf interacts with Rols [27], no such interaction has been shown for Loner. Rols is thought to physically link Duf to elements of the cytoskeleton namely D-Titin, a muscle structural protein [30] and Myoblast city (Mbc), the *Drosophila* Dock180 homolog [25], [31], in addition to replenishing Duf at the surface of the precursor thereby sustaining fusion [25]. Arf6 has been shown to perform several roles including the regulation of Rac, an actin regulating protein [32], [33]. Consistent with this function Rac is mislocalised in *loner* mutants [29]. It has been suggested that the Rols-Mbc and the Loner-Arf6 pathways function in parallel and converge onto Rac although more recently Dyer *et al*. have reported that the loss of Arf6 has no effect on myoblast fusion [29], [34]. While myoblast attraction and fusion have been suggested to be mediated by interaction between Duf and SNS [35], downstream events that lead to changes in the cytoskeleton are still unresolved.

Given the importance of Duf during myogenesis, we asked if the intracellular domain of Duf contained any specific sites or regions that could reveal its downstream functions. Duf and Rst share significant homology in their extracellular and transmembrane regions [9], [10]. Their intracellular domains though only 15% identical, show the presence of well conserved putative signalling motifs namely, 4 putative phosphorylation sites (3 Tyrosines and 1 Serine) two of which lie in a putative autophosphorylation domain, a PADVI motif of unknown function and a C terminal PDZ binding domain [9]. Phosphorylation of Tyrosine residues in the intracellular domain of SNS has been shown to play an important role in myoblast fusion [36]. Also, the PDZ binding domain of Rst has been shown to play a significant role in *Drosophila* eye development [37]. Transmembrane domains have been shown to be critical for membrane fusion and lipid bilayer mixing [38]. These sites were mutated individually in addition to larger intracellular truncations in order to uncover critical functional domains of Duf. The function of these regions was addressed by assessing their ability to translocate Rols and Loner to sites of cell-cell contact in S2 cells and their ability to rescue *duf, rst* mutant embryos.

In this paper we show that the intracellular domain of Duf between amino acids 687 and 830 is essential for efficient fusion and in the translocation of both Rols and Loner. Putative signalling motifs analysed suggest that they are additive in function. This implies that Duf might have multiple downstream functions and interactors that play a role in different aspects of fusion, finally leading to the formation of a mature muscle.

Previous studies proposed that myoblast fusion is divided into two steps that are molecularly distinct. The first round of fusion leads to the formation of a bi/trinucleate precursor and requires molecules like Duf and Rst while later rounds of fusion require molecules like Rols, functions predicted by the phenotype of these mutants [9], [10], [26]–[28]. It has recently been proposed that the 2 steps in myoblast fusion may not be molecularly distinct. Instead, less frequent fusion events might occur initially followed by more frequent events in the later stages giving rise to two temporal phases of fusion and that all gene products required for the early phases are likely also required for the later phases of fusion [17]. Thus far Rols has been shown to play a role only in the second phase of fusion. Beckett and Baylies [17] have demonstrated that *loner* mutants block fusion at the precursor stage. Similarly, we show here that *rols* and *loner* single mutants block fusion at the precursor stage. In addition, we also asked if removal of both rols and *loner* (*rols, loner* double mutant) impaired fusion further. We also tested the fusion efficiency of other well characterised fusion mutants like *Drosophila* WASp interacting protein (*D-WIP*)/Verprolin 1 (*vrp1*)/Solitary (*sltr*) that block fusion after the formation of the precursor [39], [40] and blown fuse (*blow*) that occassionaly shows binucleate precursors [17], [41], in combination with *rols* and *loner*.

We find that in a *rols, loner* double mutant fusion is completely blocked and in other double mutants it is significantly compromised. Thus, the complex process of myoblast fusion appears to be tightly regulated and its efficiency depends on the simultaneous function of several genes. Our results support the view that there may not be a difference in the requirement of gene products in the early versus later phases of fusion and all fusion molecules might be involved in activating and sustaining the fusion process albeit through different mechanisms early versus later on during myogenesis.

## Results

### Duf Intracellular Domain between Amino Acid 687 and 830 Plays an Important Role in the Translocation of Rols and Loner in S2 Cells

**Figure 1:**
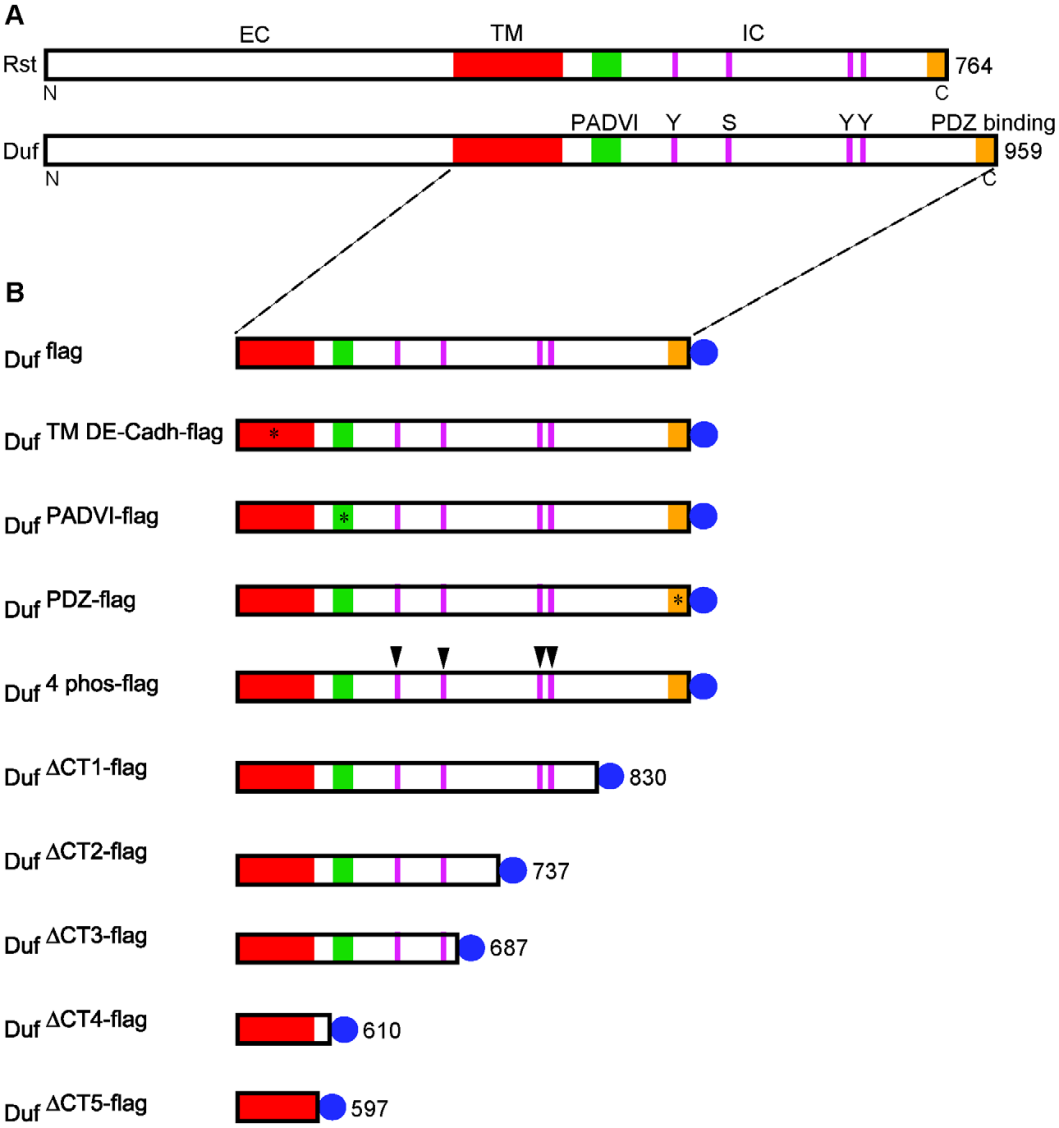
Duf mutant constructs. (A) Conserved putative signalling sites and domains between Duf and Rst. Transmembrane domain (red), PADVI domain (green) phosphorylation sites (purple and arrowheads in B) Tyr (Y) 638, Ser (S) 680, Tyr (Y) 810 and Tyr (Y) 814, PDZ binding domain (orange). (B) Duf transmembrane (TM) and intracellular (IC) domains depicting the individual mutant constructs. Asterisk indicates mutated transmembrane domain (DE-Cadh/Sema-1a) and arrowheads indicate mutated putative phosphorylation sites. Duf ^ΔCT1-flag^, Duf ^ΔCT2-flag^, Duf ^ΔCT3-flag^, Duf ^ΔCT4-flag^ and Duf ^ΔCT5-flag^ are truncated forms at amino acids 830, 737, 687, 610 and 597 respectively. All constructs were tagged with the Flag epitope (blue) at the C terminus.

In order to delineate intracellular and transmembrane regions of Duf that are critical for its function, putative signalling motifs and regions conserved with Rst were mutated using site directed mutagenesis as indicated in Fig. 1B. Here, Duf function was tested by assaying for the translocation of Rols and Loner to sites of cell-cell contact in S2 cells. All constructs were tagged with the Flag epitope at the C terminus. The transmembrane domain of Duf was replaced with that of DE Cadherin (DE Cadh)/Shotgun (Shg) that has been shown to play an important role in cell adhesion during *Drosophila* epithelial morphogenesis [42] (Duf ™^ DE-Cadh-flag^) and Semaphorin 1a (Sema-1a) that is involved in axon guidance [43] (Duf ™^ Sema-1a-flag^). The transmembrane domains of DE-Cadh and Sema-1a have stretches of similar and dissimilar amino acid sequences respectively compared to that of Duf (Supplementary information file S1). Adjacent to the transmembrane domain is a conserved series of amino acids forming a PADVI domain the function of which is unknown. This was mutated to DVPAI (Duf ^PADVI-flag^). Four putative phosphorylation sites namely, one Serine (Ser 680) and three Tyrosine (Tyr 637, 810 and 814) residues were mutated to Alanine (Ala) in a single construct (Duf ^4phos-flag^). Tyr 810 and 814 lie in a putative autophosphorylation domain. A PDZ binding motif THV, at the extreme C terminus was mutated to GAG (Duf ^PDZ-flag^). In addition, we addressed if larger regions of the intracellular domain were involved in any specific functions by generating three truncated forms of Duf. These were named Duf ^ΔCT1-flag^, Duf ^ΔCT2-flag^ and Duf ^ΔCT3-flag^ that lacked the intracellular region after amino acids 830, 737 and 687 removing 35%, 60% and 75% of the intracellular domain, respectively. Each of these mutant constructs was analysed individually.

**Figure 2:**
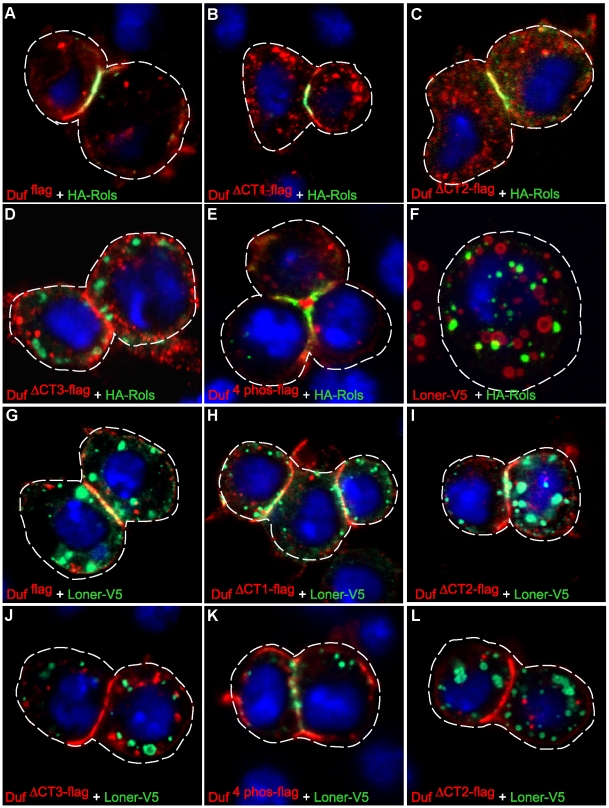
Region between amino acids 687 and 830 is important for translocation of Rols and Loner under homotypic conditions. S2 cells were co transfected with Flag tagged wild type and mutant Duf, detected with anti-Flag (red) and HA-Rols detected with anti-HA (green) (A–E) or Loner-V5 detected with anti V5 (green) (G–L). Wild type Duf ^flag^ and Duf ^ΔCT1-flag^ translocate both Rols (A and B) and Loner (G and H) to points of cell contact. Duf ^ΔCT2-flag^ and Duf ^4phos-flag^ translocate Rols (C and E) but Loner only 70% of the time (I and K). 30% of the time they are unable to translocate Loner to points of contact (compare I and L). Duf ^ΔCT3-flag^ is unable to translocate Rols (D) and Loner (J). Rols and Loner puncta do not colocalise (F). Dashed lines indicate cell outlines.

Previous studies have shown that Duf dependent translocation of Rols and Loner can be reproduced in S2 cells that adhere to each other under homotypic conditions [25], [29]. To analyse the regions of Duf required for the translocation of Rols and Loner, S2 cells were co-transfected with plasmids that expressed Flag epitope tagged wild type Duf or Duf mutants and HA epitope tagged Rols (HA-Rols) or V5 epitope tagged Loner (Loner-V5). As reported previously [25], we find that full length wild type Duf (Duf ^flag^) is enriched at the point of cell-cell contact and Rols and Loner translocate to these points of cell contact in a Duf dependent manner (Fig. 2A and G). Similarly, the mutant forms of Duf, Duf ™^ DE-Cadh-flag^, Duf ™^ Sema-1a-flag^, Duf ^PADVI-flag^, Duf ^PDZ-flag^ and Duf ^ΔCT1-flag^ are also able to translocate both Rols and Loner to sites of adhesion (Fig. 2B,H and supplementary Fig. S1A–H). Duf ^ΔCT2-flag^ and Duf ^4phos-flag^ are also able to translocate Rols to the site of adhesion (Fig. 2C and E) but translocate Loner only in 70% of the cells analysed (Fig. 2I and K). In the remaining 30% of the cells Loner is not detectable at the site of adhesion (compare Fig. 2K and L). This differential ability of Duf ^ΔCT2-flag^ and Duf ^4phos-flag^ to efficiently translocate Rols but not Loner, under homotypic conditions, might be reflective of different requirements for the translocation of Rols versus Loner. Heterotypic S2 assays that tested the ability of the Duf mutant constructs to translocate Rols and Loner in response to interaction between Duf and Sns expressing cells, confirmed the results described above although the enrichment of Rols and Loner at the site of cell-cell contact was not as robust as under homotypic conditions (Fig. S2). Interestingly, Duf ^ΔCT2-flag^ and Duf ^4 phos-flag^ failed to translocate Loner to sites of cell-cell contact (Fig. S2K,L,S,T). Cells co-transfected with Rols and Loner show that while both proteins are present as cytoplasmic foci, Loner foci are larger compared to Rols foci and the two do not colocalise (Fig. 2F) as has also been shown by Chen et al., [29]. Duf ^ΔCT3-flag^ fails to translocate both Rols and Loner under both homotypic and heterotypic conditions (Fig. 2D, J and Fig. S2M–P). These results indicate that the region between amino acid 687 and 830 of the intracellular region of Duf performs a critical function in the translocation of Rols and Loner to sites of cell adhesion. Rols is similarly enriched *in vivo* at points of FCM-precursor/myotube contact in embryos rescued with Duf ^flag^, Duf ^ΔCT1-flag^, Duf ^ΔCT2-flag^ and Duf ^4 phos-flag^ (arrow) but not with Duf ^ΔCT3-flag^ (arrowhead) expressed under 24B-Gal4 and Dmef2-Gal4 independently (Fig. S3 and S5). This might have implications for the function of Duf during myogenesis in the embryo.

### Region between Amino Acid 687 and 830 Is Important for Interaction of Duf with Rols and Loner

**Figure 3:**
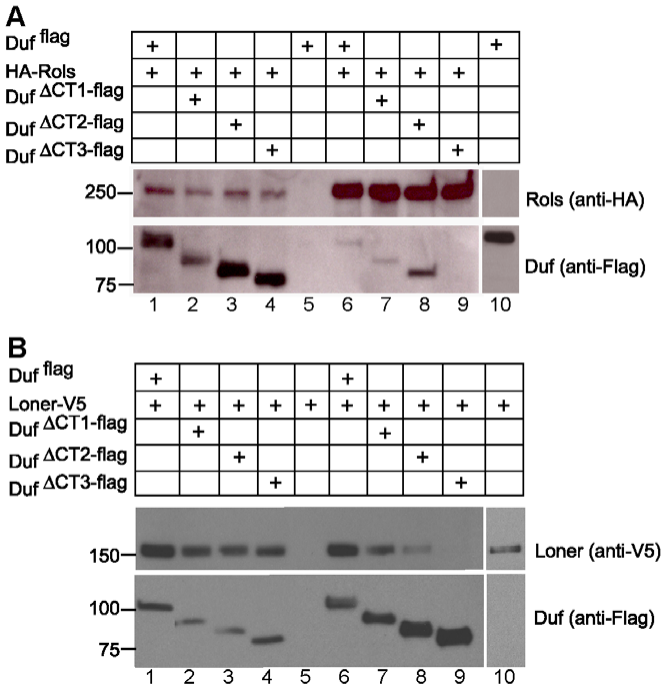
Duf intracellular domain between amino acid 687 and 830 interacts with Rols and Loner. Co immunoprecipitations (Co-IP) were performed on S2 cells co transfected with wild type Duf ^flag^, Duf ^ΔCT1-flag^, Duf ^ΔCT2-flag^, Duf ^ΔCT3-flag^ and HA-Rols or Loner-V5. Input (lanes 1–4 and 10) and Co-IP (lanes 5–9). (A) Rols-Duf complexes were pulled down with anti-HA and probed with anti-Flag to detect Duf and anti-HA to detect Rols. Wild type Duf ^flag^, Duf ^ΔCT1-flag^ and Duf ^ΔCT2-flag^ interacts with Rols (lanes 6,7 and 8) while Duf ^ΔCT3-flag^ fails to interact with Rols (lane 9). Cells transfected with Duf ^flag^ alone and immunoprecipitated with anti-HA were used as a negative control (input-lane 10, co-IP-lane 5). The input in lane 3 is ∼5 fold higher than that in lane 1. Correspondingly the IP in lane 8 is ∼5 fold higher than that in lane 6. Quantified using Image J. (B) Loner-Duf complexes were pulled down with anti-Flag and probed with anti-Flag to detect Duf and anti-V5 to detect Loner. Wild type Duf ^flag^, Duf ^ΔCT1-flag^ and Duf ^ΔCT2-flag^ interacts with Loner (lanes 6,7 and 8) while Duf ^ΔCT3-flag^ fails to interact with Loner (lane 9). Cells transfected with Loner-V5 alone and immunoprecipitated with anti-Flag were used as a negative control (input-lane 10, co-IP-lane 5).

We then addressed if the ability of Duf to translocate Rols and Loner was indicative of its physical interaction with Rols and Loner. To test this, co-immunoprecipitation assays were performed on S2 cells co-transfected with Flag epitope tagged Duf constructs and either HA-Rols or Loner-V5. Duf-Rols and Duf-Loner complexes were pulled down and individual proteins were detected on a western blot. Consistent with the immunofluorescence results obtained from S2 cells, Duf ^flag^, Duf ^ΔCT1-flag^ and Duf ^ΔCT2-flag^ interact with both Rols (Fig. 3A, lanes 6, 7 and 8) and Loner (Fig. 3B, lanes 6, 7 and 8) while Duf ^ΔCT3-flag^ fails to interact with either (Fig. 3A and B, lane 9). Duf ^4 phos-flag^ also interacts with Loner (data not shown). Thus, the breakpoints of Duf ^ΔCT2-flag^ and Duf ^ΔCT3-flag^ delineate a region in the intracellular domain (between amino acids 687 and 830) that is important for the interaction of Duf with Rols and Loner. We conclude that the same region of Duf is required for the translocation of Rols and Loner to sites of cell adhesion, and also for interaction with Rols and Loner.

### Different Intracellular Regions of Duf Function Additively for Efficient Myoblast Fusion

**Figure 4:**
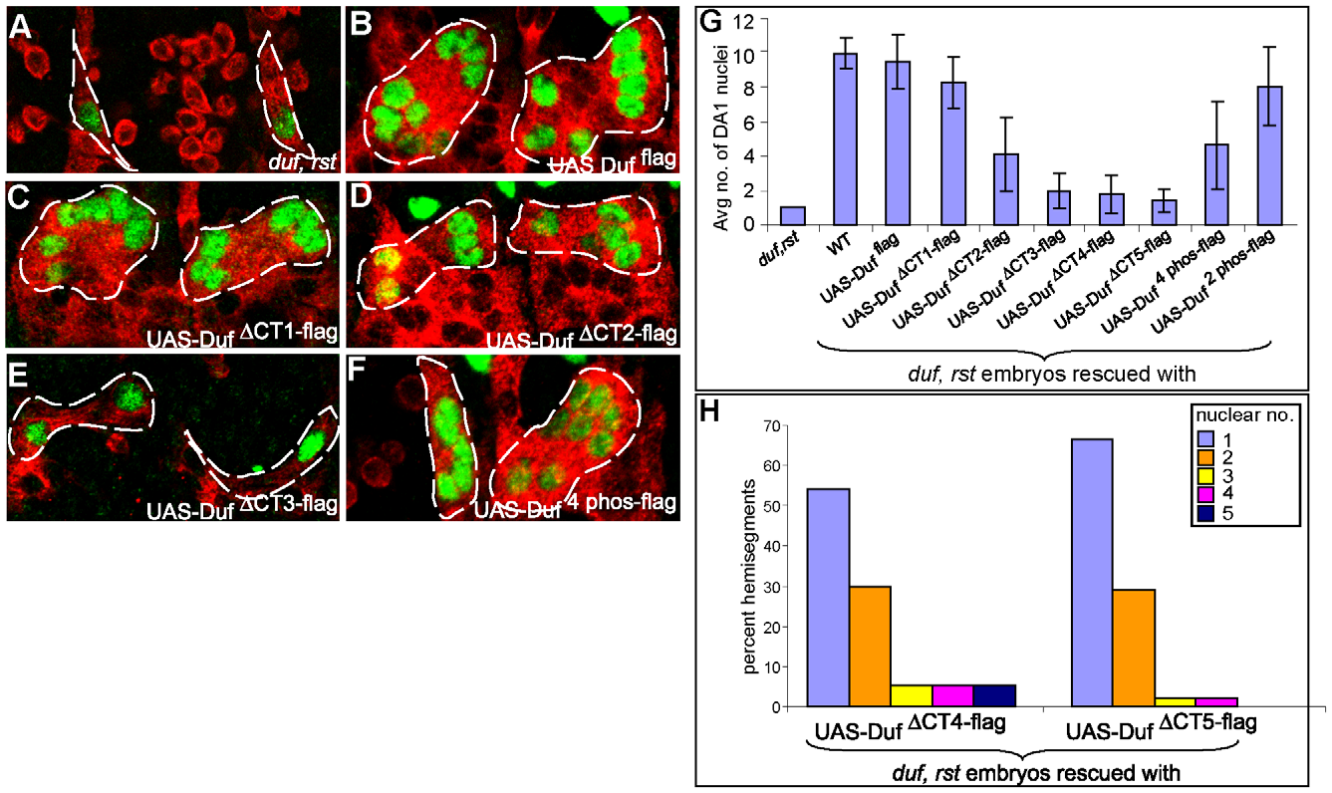
Regions in Duf intracellular domain function additively for efficient fusion. Late stage 15 DA1 muscles labelled with anti-MHC (red) and anti-eve (green). Fusion is completely blocked in the *duf, rst* mutant (A). UAS transgenic constructs driven by 24B Gal4. UAS-Duf ^flag^ (B) and UAS-Duf ^ΔCT1-flag^ (C) are able to rescue the *duf, rst* mutant. UAS- Duf ^ΔCT2-flag^ and UAS-Duf ^4phos-flag^ rescue the *duf, rst* mutant only partially (D and F). UAS- Duf ^ΔCT3-flag^ is unable to rescue the *duf, rst* mutant beyond the initial stage of fusion. (G) Average nuclear number per DA1 muscle (40 hemisegments, A2–5, 10 embryos each) in embryos rescued with UAS-Duf ^flag^ and UAS Duf mutant constructs in comparison with wild type (WT) and the *duf, rst* mutant. (Students t-test P<0.001) (H) Distribution of nuclear numbers per hemisegment in *duf, rst* embryos rescued with UAS-Duf ^ΔCT4-flag^ and UAS-Duf ^ΔCT5-flag^.

In order to delineate putative signalling motifs or regions critical for Duf function during myogenesis, Duf ^flag^ and all the mutant Duf forms listed in Fig. 1B were tested for their ability to rescue the *duf, rst* mutant phenotype. The efficiency of rescue was quantified by counting the number of nuclei in the large dorsal DA1 muscle using antibodies against the DA1 identity marker, Eve. As reported previously there is a complete block in myoblast attraction and fusion in the *duf, rst* mutant [9], [10]. Uni-nucleate FCs form mini muscles surrounded by several unfused FCMs with randomly oriented filopodia, indicative of a lack of attraction between FCs and FCMs (Fig. 4A). The reintroduction of untagged full length Duf (data not shown), Duf ^flag^, Duf ™^ DE-Cadh-flag^, Duf ™^ Sema-1a-flag^, Duf ^PADVI-flag^ and Duf ^PDZ-flag^ using a muscle specific driver, 24B Gal4, restores FCM attraction and myoblast fusion giving rise to a wild type (WT) DA1 muscle in every hemisegment with average nuclear numbers of 9.50±1.56, 9.78±0.91, 9.96±0.75, 8.40±1.45 and 8.43±1.43 respectively, as summarised in Tables 1 and S1, at stage 15 of embryonic development (Fig. 4B, G and supplementary Fig. S4). Duf ^ΔCT1-flag^ that is able to translocate and interact with Rols and Loner (Fig. 2B, H and Fig. 3) is also able to successfully restore myoblast attraction and fusion up to an average nuclear number of 8.30±1.49 (Fig. 4C and G) compared to the wild type DA1 nuclear number of 9.50±1.56 (Fig. 4G). Interestingly, the expression of Duf ^ΔCT2-flag^ and Duf ^4phos-flag^ only partially restores fusion to an average nuclear number of 4.07±2.15 and 4.61±2.58 respectively (Fig. 4D, F and G). It is important to note that Duf ^ΔCT2-flag^ lacks 2 of the 4 phosphorylation sites mutated in Duf ^4phos-flag^. These 2 phosphorylation sites are Tyr 810 and Tyr 814 that lie in the putative autophosphorylation domain. Transgenes where both these sites are simultaneously mutated (Duf ^2 phos-flag^) and where each of these sites are individually mutated, are able to successfully rescue the *duf, rst* mutant (Fig. 4G and Supplementary Table S1). Duf ^ΔCT3-flag^ that fails to translocate and interact with both Rols and Loner (Fig. 2D, J and Fig. 3) is only able to restore the first phase of myoblast fusion up to the bi/tri-nucleate stage (Fig. 4E and G). Similar results were obtained by rescuing the *duf, rst* mutant with the founder specific Dmef2-Gal4 driver (Fig. S5).

**Table 1:** Summary of Duf intracellular domain analysis.

Construct	Rols translocation	Rols interaction	Loner translocation	Loner interaction	Rescues *duf, rst* mutant	Avg. number of DA1 nuclei (P<0.001)
Duf ^flag^	+	+	+	+	+	9.50±1.56 (P = 0.33)
Duf ^4 phos-flag^	+	nt	±	+	±	4.61±2.58
Duf ^ΔCT1-flag^	+	+	+	+	+	8.30±1.49
Duf ^ΔCT2-flag^	+	+	±	+	±	4.07±2.15
Duf ^ΔCT3-flag^	−	−	−	−	−	1.93±1.00

Rols and Loner translocation and interaction with Duf were assayed in S2 cells. DA1 nuclei in 40 embryonic hemisegments were counted in late stage 15 embryos. Average number of nuclei ± standard deviation is shown. Symbols and abbreviations: +  =  present at site of cell-cell contact, rescues *duf, rst* mutant to levels comparable to that of WT −  =  not present at site of cell-cell contact, does not rescue *duf, rst* mutant, ±  =  present only sometimes at site of cell-cell contact, partially rescues *duf, rst* mutant, nt  =  not tested.

We further investigated if the remaining 90 amino acids or 25% of the intracellular domain contributed to the function of Duf and if fusion was further compromised upon removal of this region. Truncated Duf forms used to address this were Duf ^ΔCT4-flag^ and Duf ^ΔCT5-flag^ that lacked the intracellular region beyond amino acid 610 and 597 removing 96% and 100% of the intracellular domain respectively (Fig. 1B). It has been shown previously that Duf ^ΔCT4-flag^ is able to rescue the *duf, rst* mutant up to the bi/trinucleate stage [25]. Upon the reintroduction of Duf ^ΔCT4-flag^ and Duf ^ΔCT5-flag^ into the *duf, rst* mutant, we find that the average DA1 nuclear number is similar to that shown by the expression of Duf ^ΔCT3-flag^ (Fig. 4G, Tables 1 and S1). But there is a greater percentage of hemisegments (5% v/s 2.5%) with 3 and 4 nuclei in embryos rescued with Duf ^ΔCT4-flag^ versus Duf ^ΔCT5-flag^ (Fig. 4H). Even at the terminal stages of fusion at late stage 15, *duf, rst* embryos rescued with Duf ^ΔCT5-flag^ have a maximum of 4 nuclei in their DA1 muscles while Duf ^ΔCT4-flag^ is able to rescue DA1 muscles up to 5 nuclei (Fig. 4H). While the absence of the intracellular domain does not prevent the formation of bi nucleate precursors, they are formed only 30% of the time. In 65% of hemisegments examined, fusion completely fails leading to the formation of mononucleate mini muscles. Interestingly the formation of precursors is slightly delayed in embryos rescued with Duf ^ΔCT5-flag^ (Table S2). Nevertheless, the results with Duf ^ΔCT5-flag^ formally demonstrate that formation of precursors does not require any part of the Duf intracellular domain. Expression levels of the truncated constructs were similar as shown in Fig. S6, except for Duf ^ΔCT5-flag^ which was undetectable possibly due to masking of the Flag epitope.

Consistent with the results obtained from S2 cells (Fig. 2 and 3), these data suggest that different regions and motifs of Duf intracellular domain function additively to bring about efficient myoblast attraction and fusion. Mutation of all 4 putative phosphorylation sites partially rescues the *duf, rst* myoblast fusion defect (Table 1) implying that phosphorylation of Duf might be one of several ways in which myoblast fusion is sustained and myotube growth is regulated. These sites appear to be additive in function. Serial truncations of the intracellular domain successively compromise the ability of Duf to form a mature muscle. The intracellular domain is not required for the formation of bi/trinucleate precursors, in a fraction of hemisegments examined. This suggests that the interaction of Duf with Rols and Loner is not required for its initial function but is required for increased efficiency of the process and the sustenance of myoblast fusion. The transmembrane domain might serve only to anchor Duf to the FC/myotube membrane. A change in the amino acid sequence of this domain does not affect the ability of Duf to perform its function during myoblast fusion as long as it is located at the surface of the myoblast/myotube. Surprisingly, we find that the PDZ binding domain is not required for Duf function in myoblast fusion.

### Rols and Loner Have Duf Independent Functions in the Early Stages of Myoblast Fusion

**Figure 5:**
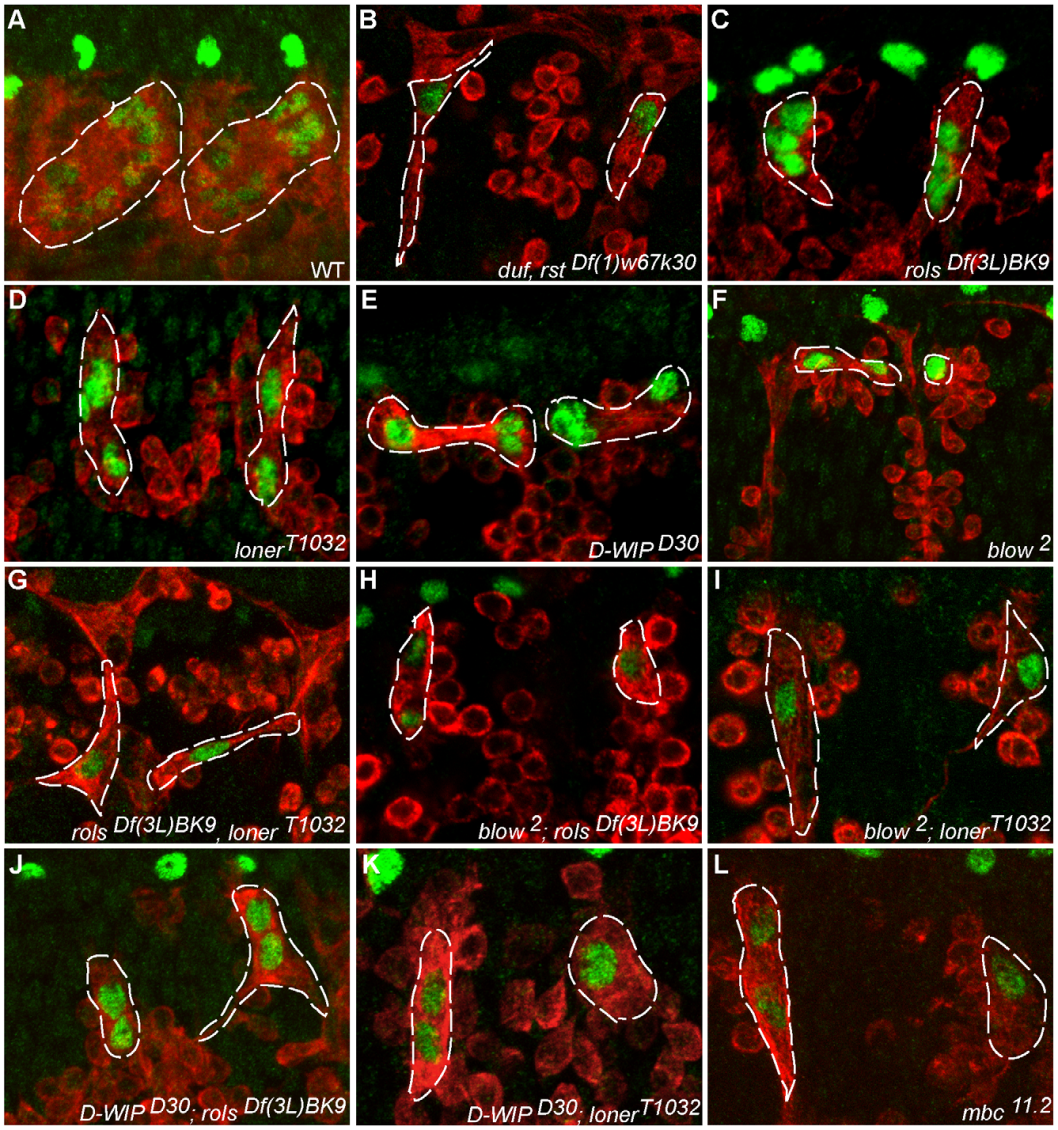
Fusion in single and double mutant backgrounds. Late stage 15-early stage 16 embryos with somatic muscles and FCM labelled with anti-MHC (red) and anti-eve (green). Wild type DA1 muscle with approximately 10 nuclei per muscle (A). *duf, rst* embryos with uninucleate DA1 muscles (B). *rols*, *loner*, *D-WIP*, *blow* and *mbc* single mutants (C–F and L). (G–K) *rols,loner*, *blow;rols*, *blow;loner*, *D-WIP;rols* and *D-WIP;loner* double mutants with reduced number of eve positive nuclei. The pericardial cells are also labelled with anti-eve but are distinguished by their brighter stain and are not surrounded by MHC positive muscle cytoplasm.

**Figure 6:**
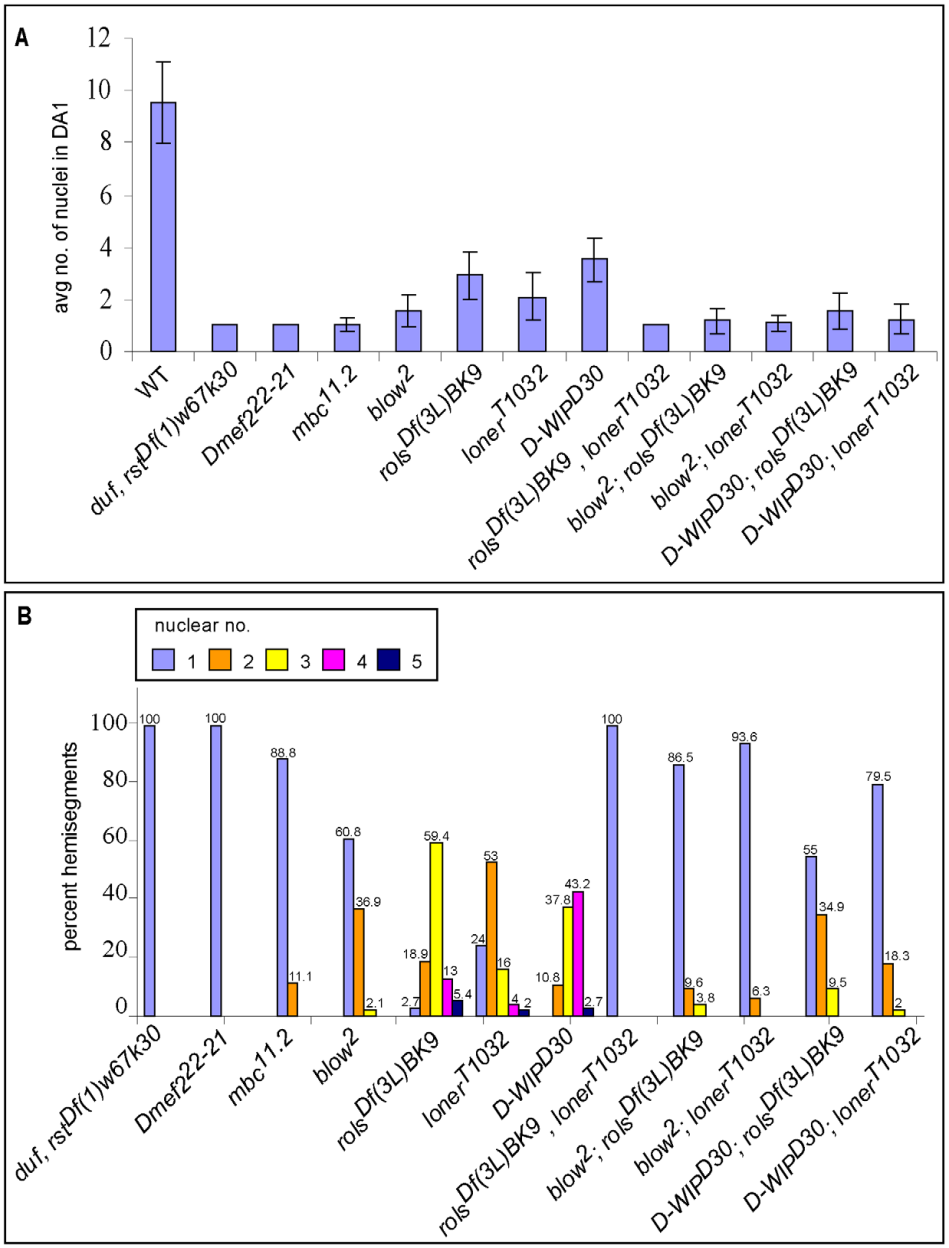
Fusion efficiency is compromised in double mutant backgrounds. Average number of Eve positive nuclei in the listed mutants, at late stage 15-early stage 16. 45 hemisegments (A2–4, 15 embryos) were counted. (A). Distribution of nuclear numbers in the listed mutants at late stage 15-early stage 16 (B). Fusion efficiency is significantly compromised in the double mutants compared to the single mutants (Students t-test P<0.001). Numbers above each bar indicate percent hemisegments for each genotype.

The present view is that different genes are involved in the early versus later stages of myoblast fusion. While it has been suggested that this might not be the case and that gene products thus far characterised to be functional in later stages of myoblast fusion might also be involved in the initial phase of fusion [23], data conclusively showing this is currently lacking. Duf/Rst have been shown to be required for the initiation of fusion [9], [10]. Although a mutation in the transcription factor myocyte-specific enhancer factor 2 (*dmef2*) also blocks the initiation of fusion, this is likely due to defects in myoblast differentiation [19]. Mbc has been characterised to be involved in the intial phase of fusion [15], [31], but recent studies have demonstrated the presence of binucleate precursors in *mbc* mutant embryos [17]. While Rols has been shown to be involved in later stages of fusion and in sustaining the fusion cycle [25]–[28], Loner has been shown to be required early on during the initial phase of fusion [17], [29].We assessed fusion efficiency by counting the number of nuclei in DA1 muscles at late stage 15-early stage 16 of embryogenesis between 13 h–14.5 h after egg laying (AEL) to minimise effects caused by a delay in fusion. The latest stage of Eve expression was chosen in order to determine as closely as possible, the terminal nuclear number in the DA1 muscle. As has been reported previously [10], [19], [44], we find that in the *duf, rst* and *D-mef2* mutants fusion is completely blocked (Fig. 5B and Fig. 6A). Consistent with data presented by Becket and Baylies [17] we find that fusion is blocked at the precursor stage in an *mbc* mutant (Fig. 5L, Fig. 6A and B) and also in *rols* and *loner* single mutants (Fig. 5C, D and Fig. 6A). To test if fusion is further impaired in the absence of combinations of such molecules and if they have functions during the initial phases of fusion, double mutants were generated by recombining the *rols* deficiency allele *rols^Df(3L)BK9^* and the loner EMS allele *loner ^T1032^*, henceforth called the *rols, loner* mutant. In addition, double mutants combining the P element excision allele *D-WIP ^D30^* and a blown fuse allele *blow ^2^* with *rols^Df(3L)BK9^* and *loner ^T1032^* were generated, henceforth called the *D-WIP;rols*, *blow*;*rols*, *D-WIP;loner* and *blow;loner* mutants. Myoblast fusion in these double mutants was compared to the single mutants.

While WT DA1 shows an average of 9.5±1.5 nuclei (Fig. 5A and Fig. 6A), the *rols* and *loner* single mutants block fusion at the bi/trinucleate stage with an average nuclear number of 2.89±0.91 and 2.11±0.91 respectively (Fig. 5C, D and Fig. 6A). Interestingly, in the *rols, loner* double mutant fusion is completely blocked (Fig. 5G and Fig. 6A) and founders remain mononucleate, similar to the *duf, rst* mutant (Fig. 5B and Fig. 6A). In both these mutants FCMs do not appear to be attracted towards the FC as indicated by the morphology of their lamellipodia which are randomly oriented (Fig. 5B and G). We also observe mononucleate Kruppel positive DO1 muscles (data not shown). While fusion in *D-WIP* embryos is blocked at the precursor stage [39], [40] (Fig. 5E and Fig. 6A) and *blow* embryos show rare fusion events upto the binucleate precursor stage [17] (Fig. 5F and Fig. 6A), fusion is significantly compromised in the *D-WIP;rols*, *blow*;*rols*, *D-WIP;loner* and *blow;loner* double mutants (Fig. 5G–K and Fig. 6A, P<0.001). There is a significant reduction in nuclear number as compared to the single mutants (Fig. 6A). While the average nuclear number is indicative of overall fusion in an embryo, we also chose to analyse these mutants by calculating the percentage of hemisegments that showed a specific number of nuclei ranging from 1–5. We find that there is a greater percentage of hemisegments with a reduced number of nuclei in the *D-WIP;rols*, *blow*;*rols*, *D-WIP;loner* and *blow;loner* double mutants as compared to the *D-WIP*, *blow*, *rols* and *loner* single mutants (Fig. 6B).

These results, summarised in Table 2, have the following implications. Thus far, besides the transcription factor Dmef2 [19], *duf* and *rst* are the only other genes that are essential for the initiation of fusion. Duf (and Rst) appears to be the limiting factor during fusion in the absence of which fusion is completely blocked [9], [10]. Importantly, we now show that Rols and Loner also function during the initial stages of myoblast fusion in a manner independent of Duf.

**Table 2:** Average number of DA1 nuclei in fusion mutants at late stage 15-early stage 16.

Fusion mutants	Avg. number of DA1 nuclei	Avg. number of DO2 nuclei
WT	9.50±1.50	10.69±1.43
*duf, rst*	1.00±0.00	1.00±0.00
*Dmef2*	1.00±0.00	1.00±0.00
*mbc*	1.07±0.26	1.08±0.31
*blow*	1.55±0.61	1.54±0.91
*rols*	2.89±0.91	2.59±0.95
*loner*	2.11±0.91	2.58±0.93
*D-WIP*	3.52±0.82	3.44±1.46
*rols, loner*	1.00±0.00	1.00±0.00
*blow; rols*	1.18±0.48	1.09±0.3
*blow; loner*	1.08±0.28	1.09±0.27
*D-WIP; rols*	1.55±0.65	1.2±0.41
*D-WIP; loner*	1.25±0.55	1.77±1.06

DA1 nuclei in 45 hemisegments (A2–4, 15 embryos each) were counted in embryos at late stage 15-early stage 16. Average number of nuclei ± standard deviation is shown. Students t-test P<0.001 for all double mutants.

## Discussion

We have shown that in order to ensure successful fusion a large part of the intracellular region of Duf is required for its function. Serial truncations of the intracellular domain reveal that the efficiency of fusion is decreased as larger regions are removed. Also, conserved putative phosphorylation signalling sites function additively resulting in efficient myoblast fusion and the formation of a mature myotube. Several parallels can be drawn from this data and that published by Kocherlakota *et al*., on the intracellular domain of the Duf ligand SNS [36]. Similar to what has been found for SNS, the PDZ binding domain is not required for the function of Duf during myoblast fusion. This is contrary to the role of this domain in the function of Rst in the developing eye [37]. While the intracellular domain of SNS is important for its function [45], the C terminal end of SNS is dispensable [36] similar to that of Duf as shown by Duf ^ΔCT1-flag^ in the Rols/Loner translocation assay in S2 cells and rescue of the fusion defect in *duf, rst* embryos. The membrane proximal intracellular regions of SNS [36] and Duf are more important for their functions. While SNS is phosphorylated on tyrosine residues [36], the ability of Duf ^4 phos-flag^ to only partially rescue the *duf, rst* mutant, implies that phosphorylation of these sites also contributes to Duf function.

Membrane anchored forms of Duf irrespective of the sequence of the transmembrane domain, appear to be sufficient for successful fusion. This suggests that the transmembrane domain of Duf does not perform any essential role or contribute to downstream signalling activity and only serves to anchor Duf to the plasma membrane. The PADVI motif, though not essential for myoblast fusion, might have a function in the context of a different tissue type that has not been tested so far. That the functions of Duf cannot be attributed to particular motifs might be a strategy utilised to ensure that normal myotube development occurs in a robust manner and compromising the function of any of these motifs singly, does not drastically affect the overall process. As has been suggested for the downstream functions of SNS [36], Duf too might transduce signals to cytoskeletal elements via its intracellular domain, to ensure successful myoblast fusion.

Previous studies proposed that myoblast fusion molecules can be categorised into those that participate in the early versus later phases of fusion [26], [41]. More recently it has been proposed that all fusion molecules are required in all fusion events [17]. Molecules like Rols and Loner have been individually shown to function in the second phase of fusion after the formation of the bi/trinucelate precursor (this study and [17], [26], [28], [29]). We have shown that removal of both *rols* and *loner* completely blocks fusion similar to the *duf, rst* mutant. Analyses of other similar double mutants demonstrate that genes involved in myoblast fusion might interact with each other to affect fusion efficiency. It is possible that what we have shown here with a few myoblast genes is true for other genes that have thus far been characterised for their role in the later stages of fusion. Such interactions have been shown for Kette/Hem/Nap1/GEX-3 and Blow [41].

We have shown that membrane anchored Duf without its intracellular domain and without any interaction with Rols and Loner, is sufficient to initiate fusion. It is possible that even in the absence of robust Duf dependent signal transduction, requirements for the formation of a bi/trinucleate precursor are met. We have also shown that Rols and Loner are required, albeit redundantly, for precursor formation or the initial phase of fusion suggesting that this “early function” of these molecules appears to be independent of Duf. We have observed this fusion defect in late stage 15-early stage 16 embryos to ensure that our observations and interpretation thereof are not due to a delay in fusion. Rols and Loner may perform different roles early versus later on during myoblast fusion. In the later phase of fusion, Rols and Loner appear to sustain fusion by interacting with and translocating Duf to the surface of the myotube [25], [29] (and this paper). As has been suggested in the case of Rols, Loner too might serve to regulate Duf at the surface of the myotube through as yet unknown mechanisms [25]. It is possible that these supposed distinct early versus late mechanisms are used in mutant conditions in an effort to overcome fusion blocks, thus leading to delayed fusion events.

## Materials and Methods

### Plasmids and Cloning

All primer sequences are listed in supplementary information file S1. Constructs in Ac5.1 for expression in S2 cells: pAc5.1A∶HA-Rols [25]. All other constructs were cloned into pAc5.1C between the EcoRV and NotI sites. Duf ^flag^ was generated by inserting the Flag-epitoe before the stop codon at the C terminus of Duf in pCI-neo and subcloned into pAC5.1A and pUAST.

Duf mutant constructs were generated using Expand High Fidelity PCR system (Roche) with primers (listed in supplementary file S1) that carried the required point mutation. Two PCR fragments were first generated using the Forward primer for the mutation + Duf-flag-R and the Reverse primer for the mutation + Duf-F. Full length Duf in pCI-neo was used as the template. The 2 fragments together served as a template for the next round of PCR using Duf-F and Duf-flag-R. This single fragment was cloned into the EcoRI and NotI sites of pCI-neo following which the full length construct was excised using NheI, blunted with Calf Intestinal Phosphatase (CIP)(New England Biolabs, NEB) and NotI and cloned into the EcoRV and NotI sites of pAc5.1C or the EcoRI (blunted with CIP) and NotI sites of pUAST. The transmembrane domain of Duf was replaced with that of DE-Cadherin and Semaphorin-1a using nested PCR. Loner-V5 was generated by cloning the Loner sequence from genomic DNA extracted from pUAST-Loner-Isoform I flies into the EcoRI and XhoI sites of pAC5.1C in frame with the V5epitope tag at the C terminus. All constructs were fully sequenced. All restriction enzymes were obtained from NEB.

### Drosophila Strains

All flystocks and crosses were maintained at 25°C. Stocks used were: yw, *rols^Df(3L)BK9^* [25], *Df(1)w^67k30^* [10], *loner^T1032^* [29], *blow^2^* [20], *mbc^D11.2^* [31], *D-mef2 ^22-21^* [19], *D-WIP^D30^* [40] and 24B Gal4 (flybase). Homozygous mutants were identified by the absence of β-galactosidase staining. Embryos were collected 13.5–14 hours AEL.

### Genetics

Transgenic flies were generated as described previously [46]. Constructs were cloned into the pUAST vector and expressed using gal4-UAS [47]. Results are representative of two independent insertions for each transgene. Rescues were performed using single copies of the UAS transgene and 24B-Gal4 in *duf, rst* mutant embryos.

### Embryo Fixation and Immunostaining

Embryos were collected at 25°C and washed in PBT (1X PBS and 0.1% Triton X-100), dechorionated in 50% bleach, rinsed in PBT, fixed in 1∶1 heptane: 4% methanol free paraformaldehyde (4%PFA with 0.1 M Hepes pH 7.4) for 15 min while shaking, devitellinated in 1∶1 heptane: methanol for 1 min and stored in 100% ethanol at −20°C. For immunostaining, embryos were rehydrated in PBT and blocked in 3% BSA-PBT. The following primary antibodies were used: mouse anti-MHC 1∶50 [48], rabbit anti-eve 1∶5000 [11], guinea pig anti-Runt 1∶2000 [49], (rabbit anti-B galactosidase (Cappel). Secondary antibodies were conjugated to Cy3 (Jackson ImmunoResearch Laboratories, Inc) or Alexa Fluor 488 (Molecular Probes). Samples were mounted in Vectashield (Jackson ImmunoResearch Laboratories, Inc) and analysed under a confocal microscope (Zeiss LSM 5 Exciter). Gut morphology was used to stage the embryos.

### Transfection and Immunostaining of S2 Cells

2×10^6^ number of S2 cells were seeded onto polylysine coated coverslips (Iwaki) in a 6 well dish at 25 degrees 24 hours prior to transfection. 0.5 ug each of Flag epitope tagged wild type Duf and the Duf mutant constructs together with 0.5 ug of either HA-Rols or Loner-V5 was co transfected into these cells using the Qiagen effectene transfection reagent. DNA to effectene ratio was maintained at 1∶20. 44 hours post transfection cells were washed in 1X PBS. Cells were fixed in 3%PFA for 30 min at room temperature (RT), washed thrice in 1X PBS followed by incubation in PBT for 15 min to permeabilise the cells. The following primary antibodies were used: rabbit anti-Flag, 1∶400 (Affinity Bio Reagents), mouse anti-V5, 1∶500 (Invitrogen) and mouse anti-HA, 1∶100 (Roche) for 1 hour at RT. Cells were washed 5 times in PBT. Secondary antibodies were conjugated to Cy3 (Jackson ImmunoResearch Laboratories, Inc) or Alexa Fluor 488 (Molecular probes). Nuclei were labeled with Hoechst 33258 (Invitrogen). Coverslips with cells were mounted in Vectashield (Jackson ImmunoResearch Laboratories, Inc). Images were obtained under a confocal microscope (Zeiss LSM 5 Exciter).

### S2 Cell Aggregation Assay

One population of S2 cells was transfected with Duf constructs and either Loner-V5 or HA-Rols. Another population was transfected with SNS. All transfections were performed as above. 36 hours post transfection, medium was washed off and cells were mixed. 24 hours later cells were fixed and stained as above. Rabbit anti-SNS was used at 1∶400.

### Co-Immunoprecipitations

Cells were transfected as above and harvested 44 hours post transfection by centrifugation at 1000 rpm and washed twice in 1X PBS. Cells were re suspended in 800 ul ice cold immunoprecipitation (IP) buffer (50 mM Tris pH 7.4, 150 mM Sodium Chloride, 2 mM EDTA, 1% Triton-X 100, 0.5% NP40 and EDTA free complete protease inhibitor tablets (Roche) and passed 6 times through a 26^1/2^G needle to lyse the cells. Cells were centrifuged at 14,000 rpm in a refrigerated centrifuge and the supernatant was collected. 50 ul of 2X Laemmli buffer was added to 50 ul of the supernatant and boiled for 5 minutes. This was used as the input. To the rest of the supernatant 60 ul of anti-Flag M2 agarose (Sigma) for the Duf-Loner IP and 100 ul of anti-HA affinity matrix (Roche) for the Duf-Rols IP was added. These were left overnight at 4 degrees on a roller. The mixture was spun down at 4°C for 1 min at 2000 rpm and washed in cold IP buffer. This was repeated four times. After the final centrifugation equal volume of 2X Laemmli buffer was added and the sample boiled for 5 min.

### Western Blot

Samples were run on a 6% SDS PAGE gel at 120 V for 2 hours. Proteins were transferred onto a PVDF membrane (Immobilon-P^SQ^, Millipore) at 90 V for 1.5 hours at 4°C. Membranes were blocked in 5% non fat milk for 1 hour at RT. The following primary antibodies were used overnight at 4°C: mouse anti-V5, 1∶1000 (Invitrogen) to detect Loner, mouse anti-HA, 1∶500 (12CA5, Roche) to detect Rols, anti-Flag, 1∶2500 (Sigma) to detect Duf. Membranes were washed with PBTw (1XPBS, 0.1% Tween) and probed with anti mouse HRP, 1∶10000 (Roche) for 1 hour at RT. Membranes were washed with PBTw (1XPBS, X% Tween) and proteins were detected using Luminol and Coumaric acid (Sigma) and Amersham Hyperfilm^ECL^.

## Supporting Information

Figure S1 Duf mutant forms that successfully translocate Rols and Loner under homotypic conditions. S2 cells were co transfected with Flag tagged wild type and mutant Duf, detected with anti-Flag (red) and HA-Rols detected with anti-HA (green) (A–D) or Loner-V5 detected with anti V5 (green)(E–H). Duf ^PDZ-flag^, Duf ^PADVI-flag^ and Duf ^TM DE-Cadh-flag^ and translocate Duf ^TM Sema 1a-flag^ are able to translocate both Rols (A–D) and Loner (E–H) to points of cell contact.
(2.29 MB TIF)

Figure S2 Region between amino acids 687 and 830 is imporant for translocation of Rols and Loner under heterotypic conditions. One population of S2 cells was co transfected with Flag tagged wild type and mutant Duf, detected with anti-Flag (green) and HA-Rols detected with anti-HA (magenta) (B,F,J,N,R) or Loner-V5 detected with anti-V5 (magenta) (D,H,L,P,T). Another population was transfected with Sns (red). Wild type Duf ^flag^ and Duf ^ΔCT1-flag^ translocate both Rols and Loner (A–H) to points of cell contact. Duf ^ΔCT2-flag^ and Duf ^4phos-flag^ translocate Rols (I, J,Q,R) but not Loner (K,L,S,T). Duf ^ΔCT3-flag^ is unable to translocate Rols (M,N) and Loner (O,P). Dashed lines indicate cell outlines.
(7.42 MB TIF)

Figure S3 Region between amino acids 687 and 830 is important for translocation of Rols *in vivo*. Stage 15 *duf, rst* embryos rescued with the indicated Duf constructs. FCM and muscles labeled with anti-Titin (red) and anti-Rols (green). Arrow indicates Rols at the site of FCM-precursor/myotube contact (arrow in B, D, F, H, L). Rols is not enriched at the point of FCM-muscle/precursor contact in embryos rescued with Duf ^ΔCT3-flag^ (J, arrowhead).
(4.92 MB TIF)

Figure S4 Duf mutant forms that successfully rescue the *duf, rst* mutant. Stage 15 DA1 muscles labelled with anti-MHC (red) and anti-eve (green). UAS transgenic constructs Duf ^TM DE Cadh-flag^ (A), Duf ^TM Sema 1a-flag^ (B), Duf ^PADVI-flag^ (C) and Duf ^PDZ-flag^ (D) driven by 24B Gal4 are able to rescue the *duf, rst* mutant.
(3.99 MB TIF)

Figure S5 Rescue of the *duf, rst* mutant using a founder specific driver and the localization of Rols *in vivo*. (A) Average nuclear number per DA1 muscle in embryos rescued with UAS-Duf ^flag^ and UAS Duf mutant constructs expressed under Dmef2-Gal4, in comparison with wild type (WT) and the *duf, rst* mutant. (B–K) Stage 15 embryos labeled with anti-DTitin (red) and anti-Rols (green). Arrow indicates Rols at the site of FCM-precursor/myotube contact. Rols does not localize to the site of fusion in *duf, rst* embryos rescued with Duf ^ΔCT3-flag^ (H,I, arrowhead). In B–G the FCM are below the plane of focus.
(3.52 MB TIF)

Figure S6 Expression levels of Duf truncations. Flag tagged Duf transgenes were over expressed under daughterless-GAL4 at 25°C. Western blot was performed on extracts from these embryos and probed with anti-Flag, to detect Duf. Tubulin was used as a loading control. The red asterisk indicates the relevant band for each construct. All constructs were expressed at similar levels except UAS-Duf ^ΔCT5-flag^, which was undetectable possibly due to masking of the Flag epitope.
(1.41 MB TIF)

Table S1 Average number of nuclei in DA1 upon rescue of the *duf, rst* mutant
(0.02 MB DOC)

Table S2 Fusion profile of *duf, rst* mutant embryos rescued with UAS-Duf ^ΔCT5-flag^
(0.02 MB DOC)

Supplementary Information File S1 Sequence information of primers used for mutagenesis and comparison of transmembrane domains of Duf, DE-Cadherin and Semaphorin-1a
(0.03 MB DOC)
