# Supplementary figures and images for: The Intracellular Domain of Dumbfounded Affects Myoblast Fusion Efficiency and Interacts with Rolling Pebbles and Loner
Source: PLoS One. 2010 Feb 23;5(2):e9374. doi: 10.1371/journal.pone.0009374 (PMC2826419; doi:10.1371/journal.pone.0009374)

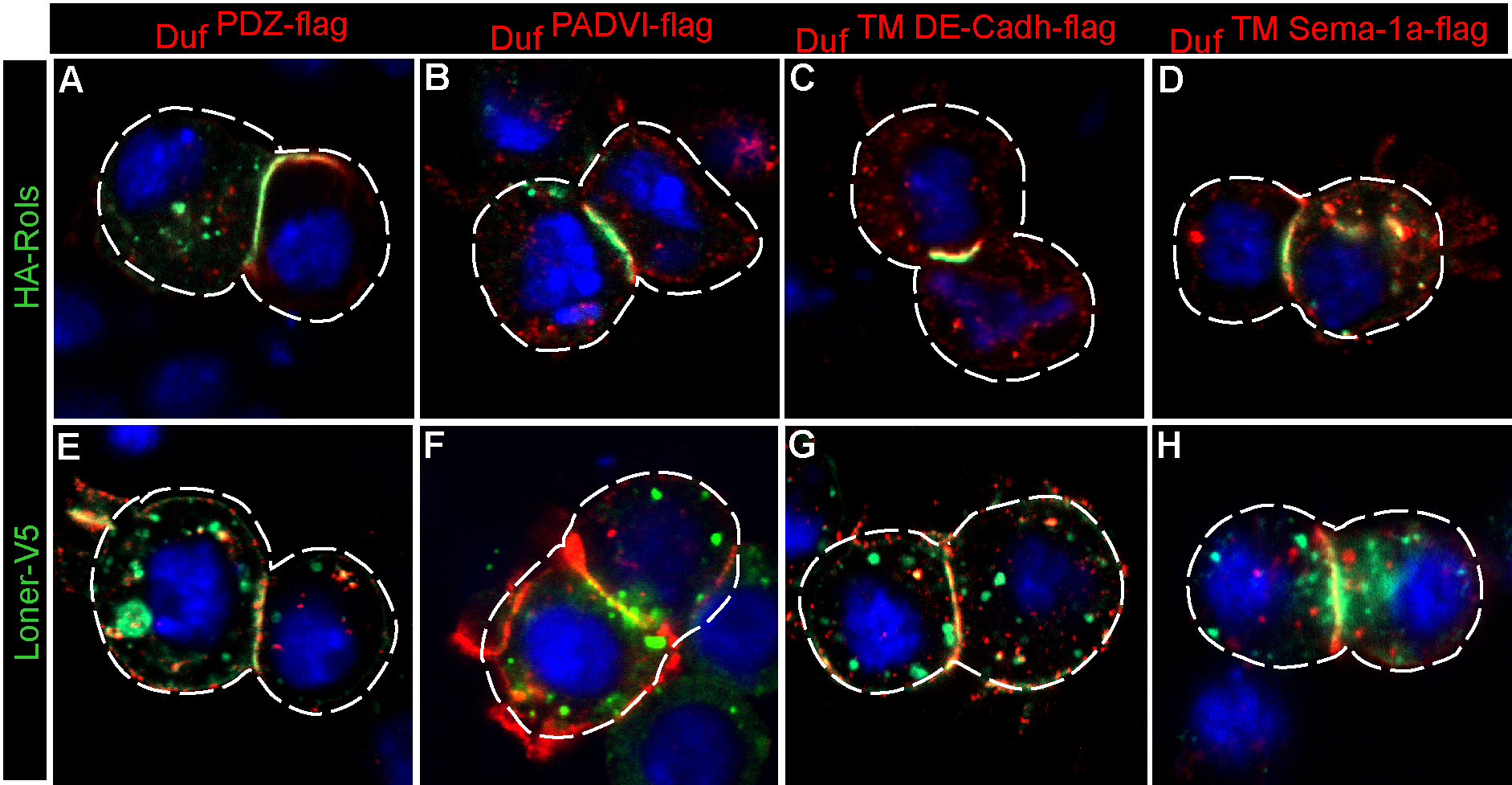

Supplement: Figure S1 — Duf mutant forms that successfully translocate Rols and Loner under homotypic conditions. S2 cells were co transfected with Flag tagged wild type and mutant Duf, detected with anti-Flag (red) and HA-Rols detected with anti-HA (green) (A–D) or Loner-V5 detected with anti V5 (green)(E–H). Duf PDZ-flag, Duf PADVI-flag and Duf TM DE-Cadh-flag and translocate Duf TM Sema 1a-flag are able to translocate both Rols (A–D) and Loner (E–H) to points of cell contact. (2.29 MB TIF) [file pone.0009374.s001.tif]

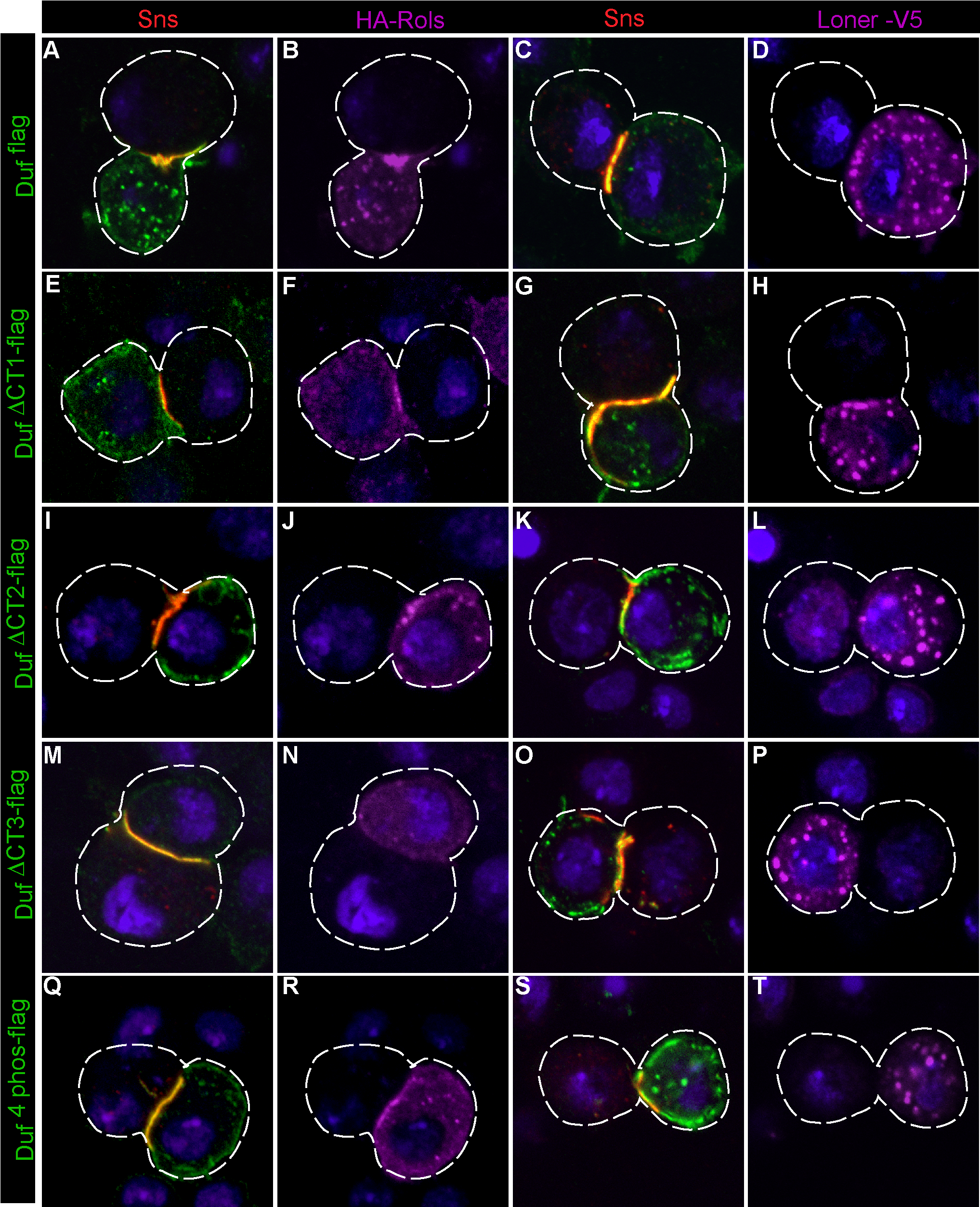

Supplement: Figure S2 — Region between amino acids 687 and 830 is imporant for translocation of Rols and Loner under heterotypic conditions. One population of S2 cells was co transfected with Flag tagged wild type and mutant Duf, detected with anti-Flag (green) and HA-Rols detected with anti-HA (magenta) (B,F,J,N,R) or Loner-V5 detected with anti-V5 (magenta) (D,H,L,P,T). Another population was transfected with Sns (red). Wild type Duf flag and Duf ΔCT1-flag translocate both Rols and Loner (A–H) to points of cell contact. Duf ΔCT2-flag and Duf 4phos-flag translocate Rols (I, J,Q,R) but not Loner (K,L,S,T). Duf ΔCT3-flag is unable to translocate Rols (M,N) and Loner (O,P). Dashed lines indicate cell outlines. (7.42 MB TIF) [file pone.0009374.s002.tif]

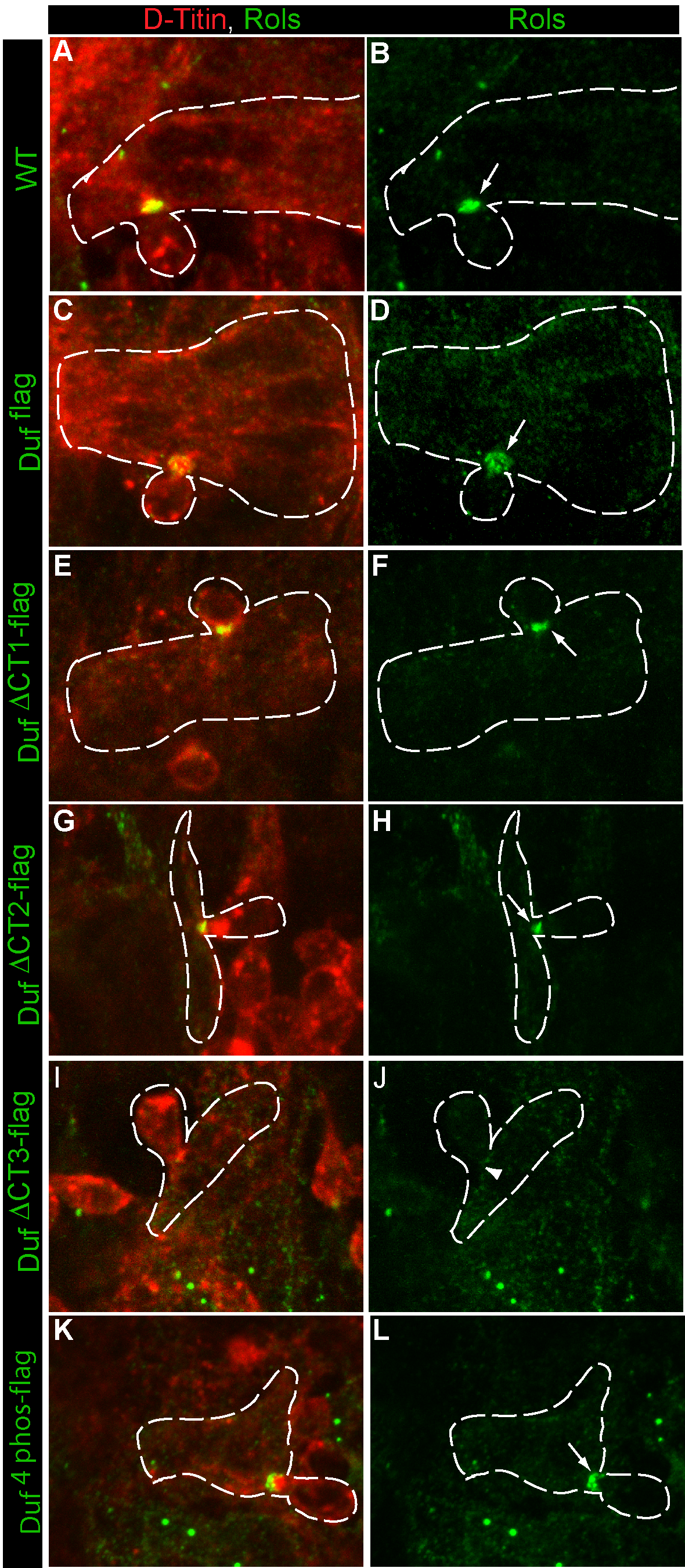

Supplement: Figure S3 — Region between amino acids 687 and 830 is important for translocation of Rols in vivo. Stage 15 duf, rst embryos rescued with the indicated Duf constructs. FCM and muscles labeled with anti-Titin (red) and anti-Rols (green). Arrow indicates Rols at the site of FCM-precursor/myotube contact (arrow in B, D, F, H, L). Rols is not enriched at the point of FCM-muscle/precursor contact in embryos rescued with Duf ΔCT3-flag (J, arrowhead). (4.92 MB TIF) [file pone.0009374.s003.tif]

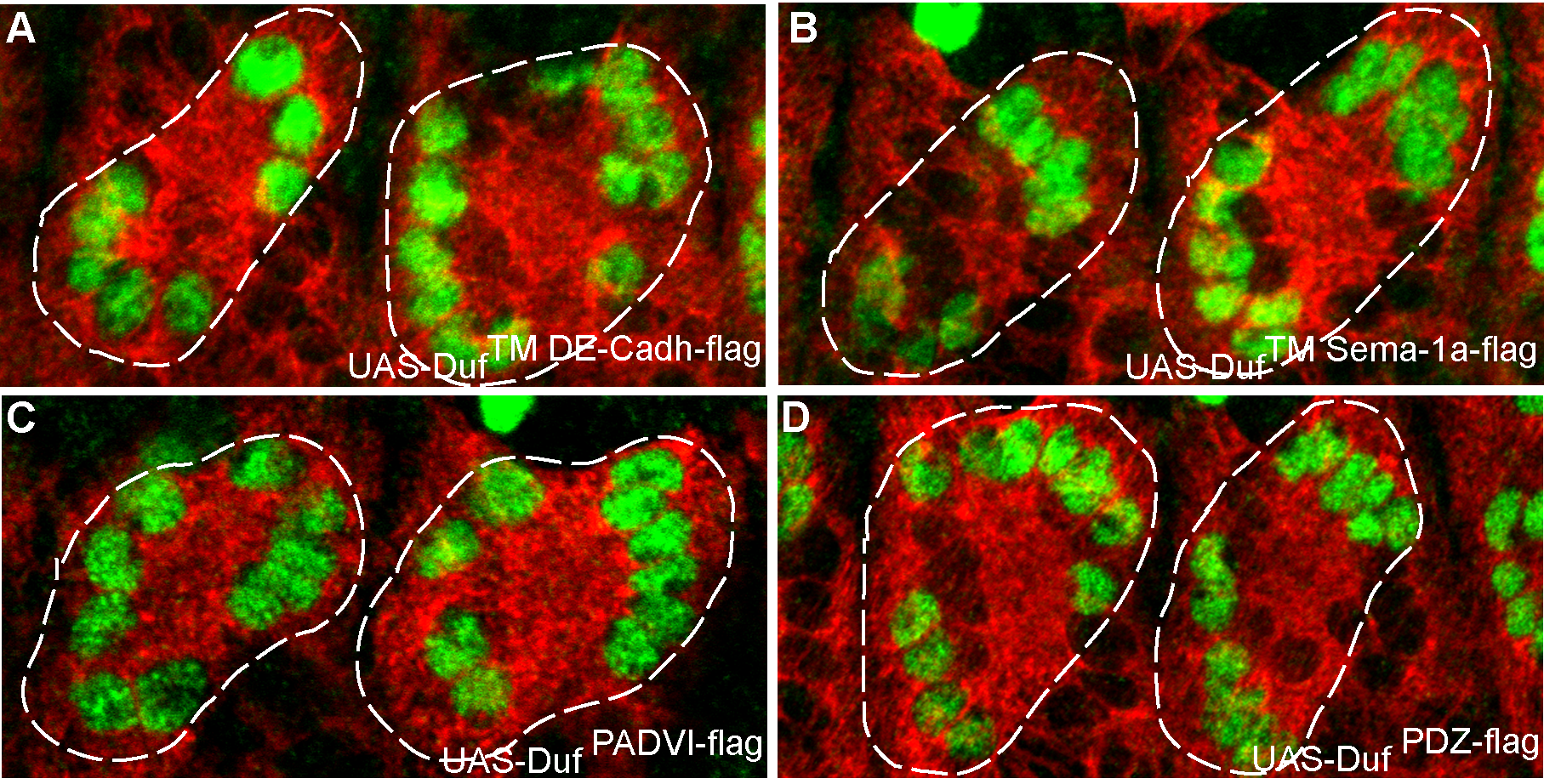

Supplement: Figure S4 — Duf mutant forms that successfully rescue the duf, rst mutant. Stage 15 DA1 muscles labelled with anti-MHC (red) and anti-eve (green). UAS transgenic constructs Duf TM DE Cadh-flag (A), Duf TM Sema 1a-flag (B), Duf PADVI-flag (C) and Duf PDZ-flag (D) driven by 24B Gal4 are able to rescue the duf, rst mutant. (3.99 MB TIF) [file pone.0009374.s004.tif]

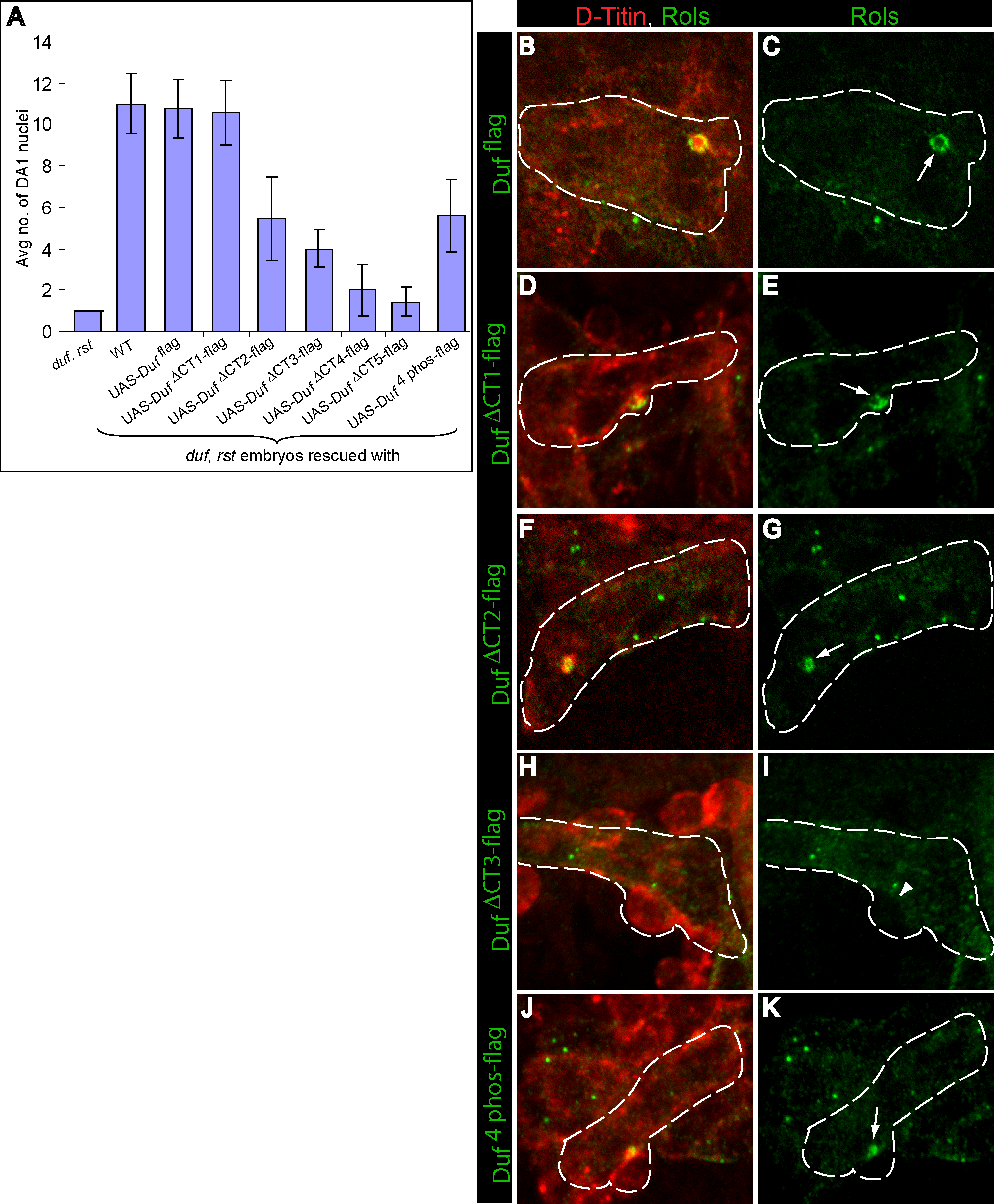

Supplement: Figure S5 — Rescue of the duf, rst mutant using a founder specific driver and the localization of Rols in vivo. (A) Average nuclear number per DA1 muscle in embryos rescued with UAS-Duf flag and UAS Duf mutant constructs expressed under Dmef2-Gal4, in comparison with wild type (WT) and the duf, rst mutant. (B–K) Stage 15 embryos labeled with anti-DTitin (red) and anti-Rols (green). Arrow indicates Rols at the site of FCM-precursor/myotube contact. Rols does not localize to the site of fusion in duf, rst embryos rescued with Duf ΔCT3-flag (H,I, arrowhead). In B–G the FCM are below the plane of focus. (3.52 MB TIF) [file pone.0009374.s005.tif]

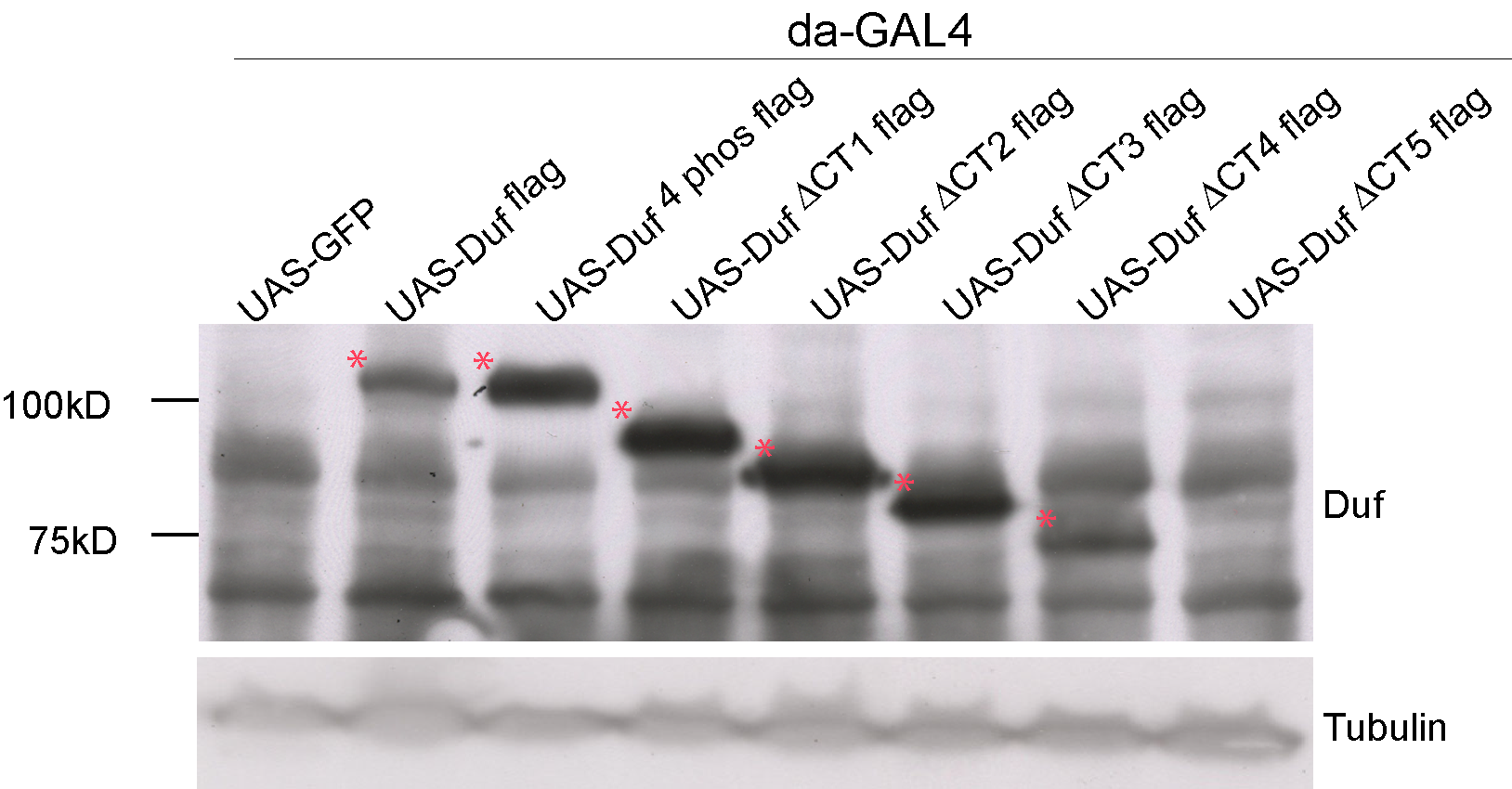

Supplement: Figure S6 — Expression levels of Duf truncations. Flag tagged Duf transgenes were over expressed under daughterless-GAL4 at 25°C. Western blot was performed on extracts from these embryos and probed with anti-Flag, to detect Duf. Tubulin was used as a loading control. The red asterisk indicates the relevant band for each construct. All constructs were expressed at similar levels except UAS-Duf ΔCT5-flag, which was undetectable possibly due to masking of the Flag epitope. (1.41 MB TIF) [file pone.0009374.s006.tif]
