# Supplementary material for: The Intracellular Domain of Dumbfounded Affects Myoblast Fusion Efficiency and Interacts with Rolling Pebbles and Loner
Source: PLoS One. 2010 Feb 23;5(2):e9374. doi: 10.1371/journal.pone.0009374 (PMC2826419; doi:10.1371/journal.pone.0009374)
Supplement: Table S1 — Average number of nuclei in DA1 upon rescue of the duf, rst mutant (0.02 MB DOC) [file pone.0009374.s007.doc]

Table S1. Average number of nuclei in DA1 upon rescue of the *duf, rst* mutant

| Construct | Rescues *duf, rst* mutant | Avg. number of DA1 nuclei |
| --- | --- | --- |
| Duf ^TM DE-Cadh-flag^ | + | 9.78 ± 0.91 |
| Duf ^TM Sema 1a-flag^ | + | 9.96 ± 0.75 |
| Duf ^PDZ-flag^ | + | 8.43 ± 1.43 |
| Duf ^PADVI-flag^ | + | 8.40 ± 1.45 |
| Duf ^2 phos-flag^ | + | 8.02 ± 2.26 |
| Duf ^Tyr637-flag^ | + | 8.20 ± 1.38 |
| Duf ^Ser680-flag^ | + | 7.90 ± 1.41 |
| Duf ^Tyr810-flag^ | + | 8.25 ± 1.29 |
| Duf ^Tyr814-flag^ | + | 8.00 ± 1.36 |
| Duf ^ΔCT4-flag^ | - | 1.78 ± 1.13 (P<0.001) |
| Duf ^ΔCT5-flag^ | - | 1.39 ± 0.64 (P<0.001) |

DA1 nuclei in 45 embryonic hemisegments were counted in late stage 15 embryos. Average number of nuclei ± standard deviation is shown. Symbols and abbreviations: + = rescues the *duf, rst* mutant comparable to WT, - = does not rescue the *duf, rst* mutant. Students t-test P<0.05 except where mentioned.
