# Supplementary material for: The Intracellular Domain of Dumbfounded Affects Myoblast Fusion Efficiency and Interacts with Rolling Pebbles and Loner
Source: PLoS One. 2010 Feb 23;5(2):e9374. doi: 10.1371/journal.pone.0009374 (PMC2826419; doi:10.1371/journal.pone.0009374)
Supplement: Table S2 — Fusion profile of duf, rst mutant embryos rescued with UAS-Duf ΔCT5-flag (0.02 MB DOC) [file pone.0009374.s008.doc]

Table S2. Fusion profile of *duf, rst* mutant embryos rescued with UAS-Duf ^ΔCT5-flag^

| Stage | WT | UAS-Duf ^ΔCT5-flag^/24B-Gal4 |
| --- | --- | --- |
| 12 | 1.2 ± 0.42 | 1.00 ± 0.00 |
| 13 | 2.00 ± 1.00 | 1.14 ± 0.37 |
| 14 | 4.71 ± 1.43 | 1.2 ± 0.42 |
| 15 | 8.84 ± 1.72 | 1.41 ± 0.61 |

DA1 nuclei in 20 hemisegments were counted at each stage. Average number of nuclei ± standard deviation is shown. Formation of precursors is delayed in embryos rescued with UAS-Duf ^ΔCT5-flag^.
