# Supplementary material for: The Intracellular Domain of Dumbfounded Affects Myoblast Fusion Efficiency and Interacts with Rolling Pebbles and Loner
Source: PLoS One. 2010 Feb 23;5(2):e9374. doi: 10.1371/journal.pone.0009374 (PMC2826419; doi:10.1371/journal.pone.0009374)
Supplement: Supplementary Information File S1 — Sequence information of primers used for mutagenesis and comparison of transmembrane domains of Duf, DE-Cadherin and Semaphorin-1a (0.03 MB DOC) [file pone.0009374.s009.doc]

Supplementary Information S1

**Primer sequences used for site directed mutagenesis**: F=Forward, R=Reverse. All sequences start from the 5’ end. Mutations and Flag-epitope sequences are underlined. Overhangs and restriction enzyme sites are in lowercase.

Duf-F: aaaaaagaattcGTATGGCGGTGACTC

Duf-flag-R: aaaaaagcggccgcTTACTTGTCATCGTCATCCTTGTAGTCAACATGAGTGGCCAGAGGTCC

Duf-PADVI-F: GATGTACCAGCGATACCGGAGGCATCACGCGGCGG

Duf-PADVI-R: TATCGCTGGTACATCCATGGGCTTCTTGCGACTGCG

Duf-Tyr 638-F: TATGATGTGGAAGCCTCGGAGGCGGGC

Duf-Tyr 638-R: CGCCTCCGAGGCTTCCACATCATAGG

Duf-Ser 680-F: GATGAGCGCTTTGCGGGCGATTTCGG

Duf-Ser 680-R: ACCGAAATCGCCCGCAAAGCGCTCATCG

Duf-Tyr 810-F: GCCATCGCTGGTAATCCCTATTTAAGGACGAACTCC

Duf-Tyr 810-R: TAAATAGGGATTACCAGCGATGGCGCTAAAGCG

Duf-Tyr 814-F: GCCATCTATGGTAATCCCGCTTTAAGGACGACGAACTCC

Duf-Tyr 814-R: TAAAGCGGGATTACCATAGATGGCGCTAAA

Duf-PDZ-flag-R: aaaaaagcggccgcTTACTTGTCATCGTCATCCTTGTAGTCACCAGCACCGGCCAGAGGTCCTTTCTGC

Duf-TM-DE-Cadh-F1: aaaaaagaattcGTATGGCGGTGACTCGTTGGTTATAACATTGCTACGCGAACCGGGCAACATACCCGTTCTGTTC

Duf-TM-DE-Cadh-R1: TTTTTTAGGCCTTCGAGCGTAGCTCGCTCTTCAATTCGTTTAGCTTATCACCGCCGCGTGATGCCTCCGG

Duf-TM-DE-Cadh-F2: TCCTGTTGGCAGTGGTGCGCAAGCGACGCAGTCGCAAGAAGCCCATGCCAGCGGATGTAATACCGGAGGCATCACGCGGCG

Duf-TM-DE-Cadh-R2: GcgCACCACTGCCAACAGGATGATCAGCAGTAGCGCGAGGCATACGATGATCGCAATGATGAACAGAACGGGTATGTTGC

Duf-TM-Sema-1a-F1: AAAAAAGAATTCGTATGGCGGTGACTCGTTGGTTATAACATTGCTACGCGAACCGGGCAACATACCCGTTCTGACCCTCG

Duf-TM-Sema-1a-R1: TTTTTTAGGCCTTCGAGCGTAGCTCGCTCTTCAATTCGTTTAGCTTATCACCGCCGCGTGATGCCTCCGGTATTACATCCGC

Duf-TM-Sema-1a-F2: TCGGCTTCTTTACAGGCTACTTCCGCAAGCGACGCAGTCGCAAGAAGCCCATGCCAGCGGATGTAATACCGGAGG

Duf-TM-Sema-1a-R2: GTAGCCTGTAAAGAAGCCGACCAGCAGCGAAAAGATCGAACCGGCCAGAACGGCCATCACGAGGGTCAGAACGGGTATG

Duf-ΔCT1-flag-R: aaaaaagcggccgcTTACTTGTCATCGTCATCCTTGTAGTCGCTCGGCGGCGGCAGGGGCG

Duf-ΔCT2-flag-R: aaaaaagcggccgcTTACTTGTCATCGTCATCCTTGTAGTCCACCACCACGGCAGCACCC

Duf-ΔCT3-flag-R: aaaaaagcggccgcTTACTTGTCATCGTCATCCTTGTAGTCATCGCCACCGAAATCGCCCG

Duf-ΔCT5-flag-R: aaaaaagcggccgcTTACTTGTCATCGTCATCCTTGTAGTCGTACACGATAATAATCATTACGATC

To generate the Duf 4-phos-flag mutant, phosphorylation sites were mutated in succession.

All primers between 30 and 50 bases were TOP purified and those above 50 bases were PAGE purified.

**Amino acid sequences of transmembrane domains:**

Duf: LLVVMGSMFCVAIILMIVMIIIVY

DE-Cadherin: FIIAIIVCLALLLIILLAVV

Sema 1a: TLVMAVLAGSIFSLLVGFFTGYE
